# Comparative Study of Traveling and Standing Wave-Based Locomotion of Legged Bidirectional Miniature Piezoelectric Robots

**DOI:** 10.3390/mi12020171

**Published:** 2021-02-09

**Authors:** Jorge Hernando-García, Jose Luis García-Caraballo, Víctor Ruiz-Díez, Jose Luis Sánchez-Rojas

**Affiliations:** Microsystems, Actuators and Sensors Group, Universidad de Castilla-La Mancha, E-13071 Ciudad Real, Spain; joseluis.gcaraballo@uclm.es (J.L.G.-C.); victor.ruiz@uclm.es (V.R.-D.); joseluis.saldavero@uclm.es (J.L.S.-R.)

**Keywords:** robot, traveling wave, standing wave, piezoelectric, leg, miniature

## Abstract

The use of wave-based locomotion mechanisms is already well established in the field of robotics, using either standing waves (SW) or traveling waves (TW). The motivation of this work was to compare both the SW- and the TW-based motion of a 20-mm long sub-gram glass plate, with attached 3D printed legs, and piezoelectric patches for the actuation. The fabrication of the robot did not require sophisticated techniques and the speed of motion was measured under different loading conditions. In the case of the TW mechanism, the influence of using different pairs of modes to generate the TW on the locomotion speed has been studied, as well as the effect of the coupling of the TW motion and the first flexural vibration mode of the legs. This analysis resulted in a maximum unloaded speed of 6 bodylengths/s (BL/s) at 65 V peak-to-peak (Vpp). The SW approach also examined different modes of vibration and a speed of locomotion as high as 14 BL/s was achieved, requiring, unlike the TW case, a highly precise location of the legs on the glass supporting platform and a precise tuning of the excitation frequency.

## 1. Introduction

Leg-based terrestrial locomotion of robots has shown significant improvement in recent decades. Companies such as Boston Dynamics, Unitree or ANYbotics reflect the level of maturity. Among the different lines of research in the field, miniaturization is one the most challenging topics. Designs with a body length of about 10 cm have already been reported [1,2,3]. However, there is a trend toward insect-scale prototypes, with a length below 10 cm. Such small-scale robots with embedded sensors might be useful to explore a hazardous environment after a natural disaster, where they could help locate survivors or track the environmental levels of chemical agents. DARPA recently opened a related program to advance the development of multi-functional mm-to-cm-scale robotic platforms [4]. Another promising application of miniature robots is the achievement of locomotion on vertical and even inverted surfaces, due to the dominance of surface adhesion forces over volume forces at smaller scales [5].

A recent review by St. Pierre and Bergbreiter [6] pointed out the advances and the difficulties associated with autonomy in power, mobility and control for sub-gram robots. A clear example of success in the field is the work by the group of Robert J. Wood, with a progressive improvement in the performance and the miniaturization of the proposed robot [7,8,9,10,11,12]. This biologically inspired robot possessed a sophisticated flexure-based transmission linkage connected to the legs to induce different gait cycles. The latest report presented a sub-gram 22.5 mm structure with a high-speed locomotion of 13.9 bodylengths per second (BL/s).

In the pursuit of miniaturization and established fabrication protocols, silicon-based robots were also reported, with a performance that still needs to be improved in terms of speed of locomotion [13,14,15,16]. The reports of magnetically actuated robots, with a speed of 15 BL/s for a 3D-printed 1 mg robot, are also worth mentioning [17,18]. Thinking about locomotion mechanisms which are easy to integrate in small-scale structures, a wavelike motion of the body of the robot, with attached passive legs, is a promising approach. If the nature of the wave is progressing, a so-called travelling wave (TW), the trajectory of the tip of the legs will be elliptical [19]. Such a wave could be attained by Rayleigh wave generation [20] or by the proper mixing of two consecutive standing waves [21,22]. Recently, the locomotion of a glass structure by combining TW generation with 3D-printed passive legs was demonstrated [23]. Such an elliptical trajectory might also be achieved with the combination of two modes at a localized position [24,25], with the advantages associated with resonant excitation. If the nature of the wave is standing, a so-called vibration mode, then the trajectory of the leg tip will be linear. He et al. [26] reported a standing wave (SW) bidirectional motor for slider motion and, later, others [27,28] demonstrated the locomotion of mm-sized structures based on the same principle. The maneuverability of this last approach was also demonstrated [29].

The motivation of this work was to compare both TW- and SW-based locomotion on the same structure and for various resonant modes. A 20-mm long sub-gram glass structure, with attached 3D-printed legs, was considered. In terms of fabrication, the simplicity of the approach stands out, as it does not require sophisticated manufacturing techniques. Piezoelectric patches were used, an already well-established actuation scheme, due to their feasibility of integration. Regarding the TW mechanism, the contribution is twofold. On the one hand, the TW generation requires the mixing of two modes, and here, how different pairs of modes affect the speed of the robot was analysed. On the other hand, instead of exciting with a TW frequency much smaller than the first natural frequency of the legs to avoid coupling effects [23,30], here, the effect of this coupling was explored, by matching the two mentioned frequencies in the quest for an improvement in the locomotion. Finally, TW and SW driving mechanisms were compared, and the SW approach resulted in a speed of locomotion that was more than twice the speed of the TW generation approach, as expected, due to a higher displacement of the legs, although this had the requirement of a highly precise location of the legs on the glass structure and a precise tuning of the actuation frequency. The contributions of the article could be summed up as follows: unsophisticated fabrication protocol, guidelines for device design, and high-speed locomotion.

## 2. Design

**Figure 1:**
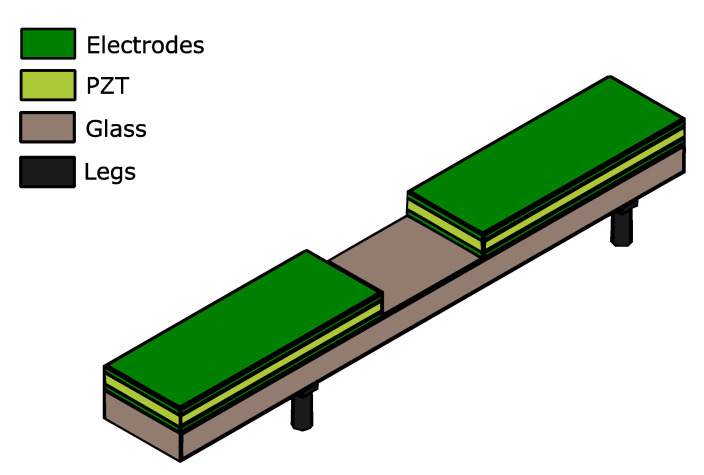
Schematic of the piezoelectric robot structure under study.

Next, considerations that need to be taken into account in the design of the devices are presented. Such a design should provide an overview of the mechanisms used to generate the waves, the influence of the vibration modes involved, and the position and the size of the legs. Figure 1 shows the type of structure under consideration. It consisted of a supporting platform of glass with a length of 20 mm, width of 3 mm, and thickness of 1 mm. Piezoelectric patches of lead zirconate titanate (PZT) were used for the actuation. The patches started at the edges of the glass and both covered a given length, to be determined by design, creating a symmetric configuration with respect to the center of the structure.

Leissa’s nomenclature was used to identify the modes of vibration (the first digit is the number of nodal lines along the length of the plate and the second digit is the number of nodal lines along the width of the plate) [31]. Two combinations of modes were studied: the pair of modes (30) and (40), named the low-order combination in what follows, and the pair of modes (50) and (60), named the high-order combination. Next, both the TW-based and the SW-based locomotion are examined.

### 2.1. TW-Based Locomotion

Here two figures of merit are considered: (i) standing wave ratio (SWR), defined as the ratio of the maximum to the minimum value of the TW envelope, which is related to the quality of the traveling wave: the closer to 1, the better the traveling wave; (ii) the average displacement of the TW envelope, named <TW>, which is associated with the speed of the wave. In the rest of the section, SWR and <TW> were taken in a central window of the total length, corresponding to 60%, in order to remove the effect of the boundary conditions at the edges.

Following the procedure detailed in reference [23], a 1D model that represented the structure actuated by the piezoelectric patches was implemented in Matlab [32]. Table 1 shows some of the parameters assumed in the model. Other parameters to consider are the following: the amplitude of the voltage applied to the patches was 10 V, the piezoelectric coefficient d31 of PZT was 180 pm/V, and the damping factor of the modes was assumed to be 0.001, corresponding to a quality factor of 500.

**Figure 2:**
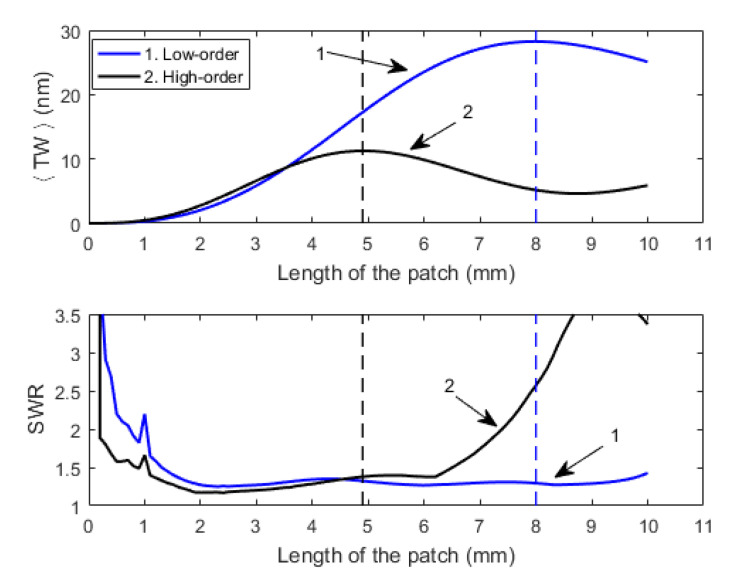
Average displacement of the traveling wave envelope (<TW>) and standing wave ratio (SWR) as a function of the length of the patch for both the low-order (blue) and the high-order (black) combinations.

Such an analysis allowed us to study the effect of the length of the patch on both the <TW> and the SWR (Figure 2). The actuation frequency of the sinusoidal signal applied to the patches and the phase shift between these signals was fixed to the mid-frequency between the frequencies of vibration of the modes under consideration and to 90°, respectively. According to the 1D model, the frequency of vibration of mode (30) was 39.8 kHz and the frequency of mode (40) was 78.1 kHz, so the actuation frequency of the TW for this pair of modes was tuned to 59 kHz. This value increased up to 161 kHz for modes (50) and (60), with frequencies of vibration of 129.1 and 193 kHz, respectively. It should be noticed that both the frequency of actuation (mid-frequency between modes) and the phase shift (90°) might be changed, maintaining the quality of the TW [23]. This means that the generation of a TW is not constrained to this particular combination, and if the frequency of actuation is varied, the achievement of a high-quality TW can be accomplished by tuning the phase shift.

As can be seen in Figure 2, there was a maximum of amplitude, <TW>, for each combination of the modes, represented by the dashed vertical lines. Such maxima also corresponded to low values of SWR. For the low-order combination, such maximums took place at a patch length of 8 mm. For the high-order combination, the size of the patch that corresponded to the maximum was 4.9 mm. In addition, a higher displacement of the TW for the combination of the modes (30) and (40) was observed, whose effect on the locomotion will be studied.

Regarding the location of the legs, as already reported in reference [23], in order to ensure an elliptical movement of the legs, they should be located in the central plateau of the TW. Therefore, as long as the legs are not close to the edges of the structure, there is ample flexibility to choose the position of the legs. Considering the length of the legs, an important variable in order to tune the intrinsic frequency of vibration of the legs, is equally interesting, assuming they resemble a beam attached to the supporting platform. The previous approach [23,30] was that the frequency of the first vibration of the legs was far above the frequencies of the modes considered for the supporting platform, in order to avoid coupling effects. However, in this paper, it was decided to investigate this coupling as a potential enhancement of the locomotion speed, and a length of leg that resulted in its fundamental flexural mode near the excitation frequency of the TW was estimated, between the two modes of vibration of the platform. For this estimation, COMSOL finite element analysis was used [33]. A 2D model of the robot was implemented, including the glass-supporting platform, the legs and the piezoelectric patches for actuation. Friction forces were neglected. A density of 1.100 kg/m^3^ and a Young modulus of 1.75 GPa were considered for the resin material of the legs. Due to uncertainty about the two previous values, the objective was to deduce a leg length whose resonance was close to the excitation frequency of the TW, not requiring an exact match. For the low-order combination, a length of 1.2 mm was estimated, with a first vibration at 52 kHz. For the high-order combination, 0.5 mm long legs were predicted, resonating at 156 kHz. To compare these resonant legs with the case where the leg fundamental resonance is high above the frequencies involved in the TW generation, two additional legs were fabricated, with lengths 0.8 mm (estimated resonance at 90.6 kHz) and 0.4 mm (estimated resonance at 194.7 kHz), for the low-order and high-order combinations, respectively. It should also be mentioned that the leg termination was a hemisphere with a radius of 0.3 mm to facilitate a more uniform contact with the surface during locomotion.

For illustration purposes, Video S1 shows the animation of the robot vibration, obtained by finite element analysis, neglecting contact. The elliptical trajectory at the tip of the legs’ originating locomotion can be noticed.

### 2.2. SW-Based Locomotion

Here, the generation of the wave corresponded to the actuation at the peak of the harmonic response of each mode of vibration. Before developing this point, it should be pointed out that, for comparison purposes, we decided to use the same device when comparing TW and SW performance. Therefore, the structure that combined modes (30) and (40) for TW generation was used for the actuation of the mode (30), as well as mode (40) in the SW robot. An equivalent method was used for modes (50) and (60). It is important to notice that the patch size and the length of the legs were already decided from the previous analysis.

Besides the two former parameters, the location of the legs on the supporting glass platform plays a crucial role in SW locomotion. As pointed out previously, there is a wide flexibility in the location of the legs of the TW robot within the central plateau of the TW envelope. Therefore, this location was based on the design of the SW bidirectional locomotion, as explained below. He et al. [26] established the criteria to be followed for SW-based systems. In the case of a slider on top of the plate, legs located at the right of a crest in the wave induce a rightward movement, while legs at the left of the crest induced a movement to the left. In our case, as a result of the contact between the legs and the static floor, movements are reversed [27]. From a mathematical point of view, a position of the structure where the ratio between the displacement and the derivative of such a displacement is positive implies a rightward movement of the robot, while a negative ratio results in a movement towards the left.

**Figure 3:**
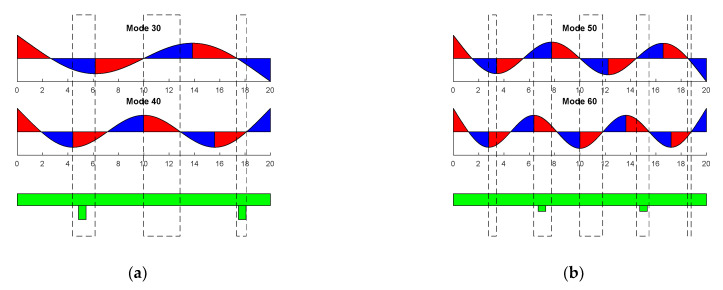
Schematics that show how to determine the location of the legs for standing wave (SW)-based locomotion, assuming a rightward movement with the lowest order mode of each combination. Vertical rectangles show the positions along the length of the device where to locate the legs. (**a**) Low-order combination. (**b**) High-order combination.

Therefore, to achieve bidirectionality, we took the same approach as previous references and searched for a position where the legs were located at the right of a crest for a given modal shape, and to the left of the crest for a subsequent modal shape. Therefore, by tuning the frequency of actuation to the resonance frequency of these modes, the direction of the movement could be changed. Figure 3 shows where to locate the legs, searching for the rightward movement induced by the lowest order modes. Red areas represent the right of a crest, and blue areas the left of a crest. The legs should be located where the blue and the red areas overlap, highlighted by the dashed rectangles in the figure. We decided to use only two of the available intervals, as shown in the figure below. For modes (30) and (40), the location at the center was avoided, with the two locations at the edges ensuring a better stability. For modes (50) and (60), the locations at the edges were avoided due to the small area used to attach the legs.

Regarding the actuation of the standing waves, i.e., the modes of vibration, an efficient excitation of a given mode can be attained by a patch distribution that covers only the regions of the surface where the sign of the second derivative of the mode shape, associated with stress on the surface, is either positive or negative [34]. As mentioned before, the patches were the same as those chosen to maximize the TW amplitude for each couple of modes, which turned out to be an intermediate length between those optimizing each SW of the same couple [23]. In that way, the performance of the same device under the two different mechanisms of locomotion could be compared. Despite not being an optimal patch, we managed to achieve the required displacement as it was corroborated by the experiments. For the actuation of either mode (40) or mode (60), the same sinusoidal signal with the frequency of the mode of vibration was applied to both patches, while for modes (30) and (50), a temporal phase shift of 180° was required between the signals applied at each patch, due to the parity of the modal shapes.

Finally, the last parameter to consider was the length of the legs. The length was also determined by the TW-based calculations. As a result, the legs for the modes (30) and (40) had 1.2 mm, and those for the modes (50) and (60), 0.5 mm. This difference in the length should be considered when analyzing the experimental results, as the horizontal displacement of the legs is proportional to the length of the legs [26].

To allow comparison with the TW-based approach, Video S2 shows the simulated displacement of the SW robot, with the associated linear movement at the tip of the legs.

To summarize all the previous information, the Table 2 below shows the operation mode for the two approaches, SW and TW. f1 and f2 are the frequencies of vibration of the couple of modes under consideration, assuming f1 < f2.

## 3. Materials and Methods

**Figure 4:**
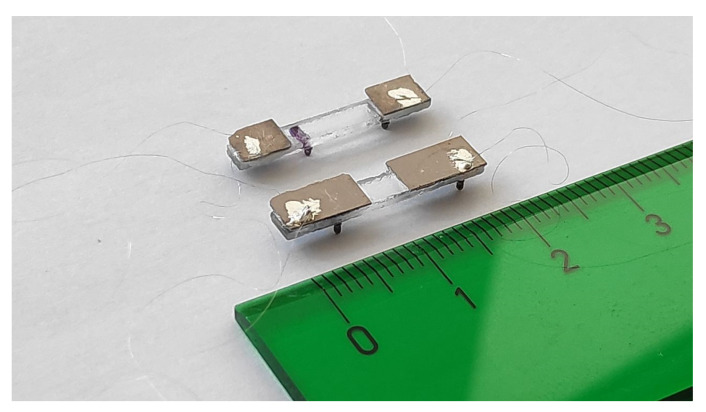
The view of a low-order combination structure, closer to the ruler, and a high-order combination structure. Ruler marked in centimeters.

A photograph of both low-order and high-order structures can be seen in Figure 4. A 20 mm long and 3 mm wide supporting platform was defined by machine-drilling from a 1 mm thick glass slide (VWR International, Radnor, PA, USA). Next two PZT patches (PIC 255 from PI Ceramic GmbH, Lederhose, Germany) with a thickness of 200 µm and a width of 3.5 mm, slightly larger than the plate to allow contact to the bottom face, were glued to the glass by means of a cyanoacrylate adhesive (Loctite, Düsseldorf, Germany). Two sizes of patch were used: a length of 5 mm for the high-order combination and a length of 8 mm for the low-order combination, according to Figure 2. Additionally, 25 µm wires were attached to the piezoelectric patches for electrical connection to the external signal generator.

U-shaped pairs of legs were 3D-printed with a stereolithography B9 Core printer (B9Creations, Rapid City, SD, USA) using the material named Black Resin (B9Creations, Rapid City, SD, USA). Each pair of legs was glued to the bottom of the supporting platform with cyanoacrylate adhesive (Loctite, Düsseldorf, Germany), following the criteria described in the design section. The shape of the legs was cylindrical, 0.6 mm diameter, with the previously discussed length: 1.2 mm for the low-order combination and 0.5 mm for the high-order combination, with a hemisphere termination at the tip in both cases. For each couple of modes, legs with a higher resonant frequency were also fabricated, to prevent plate-leg coupling, with 0.8 and 0.4 mm, respectively. The total mass of the robot was about 250 mg.

The set-up for the speed measurement consisted of two infrared LEDs separated by 10 cm, each aligned with a photodiode. With the help of a frequency counter, the set-up allowed for the measurement of the time required by the robot to travel 10 cm along a rail by tracking the light interruption events when the robot passed below the infrared LEDs. All the experiments were carried out with the robot on a glass surface. Other surfaces with similar roughness were tested, leading to similar efficiencies in terms of speed per applied volt, with slight differences in the minimum voltage at which the robot started moving and a speed could be measured.

## 4. Results and Discussion

**Figure 5:**
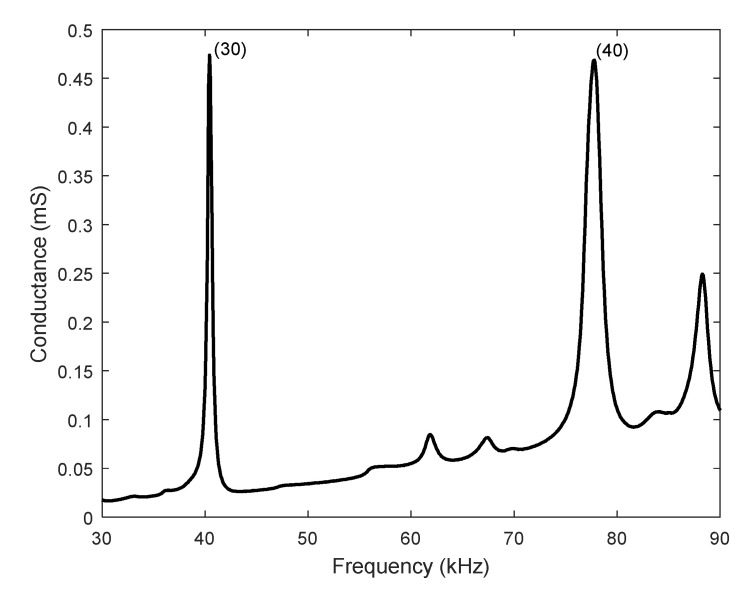
Electrical conductance vs. frequency of a structure designed for the low-order modes combination.

The analysis of the fabricated devices started with a preliminary validation by means of an impedance analyzer and an optical vibrometer. By measuring the impedance at any of the patches, the quality of the device as a resonator could be assessed, the position in frequency of the modes under study was determined and confirmed by the modal shape observed with a laser Doppler vibrometer. For the figures that followed, mode (30) was located at 39.7 kHz, mode (40) at 77.8 kHz, mode (50) at 125 kHz, and mode (60) at 180.5 kHz. Very similar values were obtained in the design of the structures, as shown in the previous section, and for the different samples prepared during this study. Figure 5 shows the electrical conductance of a robot designed for the low-order combination, with 1.2 mm legs. Two peaks can clearly be identified, corresponding to modes (30) and (40).

### 4.1. TW-Based Locomotion Results

**Figure 6:**
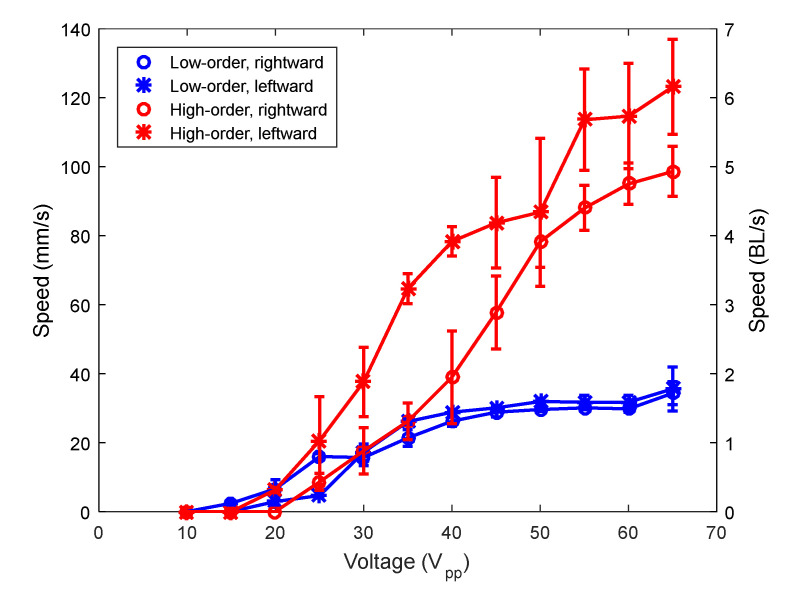
Speed of the robots (average of 5 measurements) versus applied voltage for the low-order (blue) and the high-order (red) combinations.

Here, the effect of mixing different modes, with the appropriate phase, on the locomotion performance was investigated. As mentioned before, two device designs were considered, one corresponding to the low-order combination and the other corresponding to the high-order combination. In this paper, we first compared the locomotion due to TW generated with different couples of modes and then with the SW-based robots. Figure 6 shows the speed of motion of both TW combinations versus applied voltage. Symbols represent experimental data and are joined with lines for guidance purposes. Both directions were evaluated. The phase shift between signals at the patches was fixed at either +90 or −90°, depending on the direction of movement. As mentioned before in Section 2.1, there is a certain flexibility in the selection of the excitation frequency near the midpoint between the two modes involved. This is an advantage of TW-based robots to prevent possible interaction with unwanted modes present in this interval. The frequency of actuation for the low-order and the high-order combinations was 68 and 161 kHz, respectively, after the search for the fastest speed possible by the tuning of the frequency of actuation in the interval between the combined modes. Those two experimental values were close to the exact midpoints according to the impedance spectra, at 58.7 and 152.7 kHz. A higher speed was clearly obtained for the combination of modes (50) and (60), with a maximum speed above 6 BL/s, at an applied voltage of 65 V peak-to-peak (Vpp).

**Figure 7:**
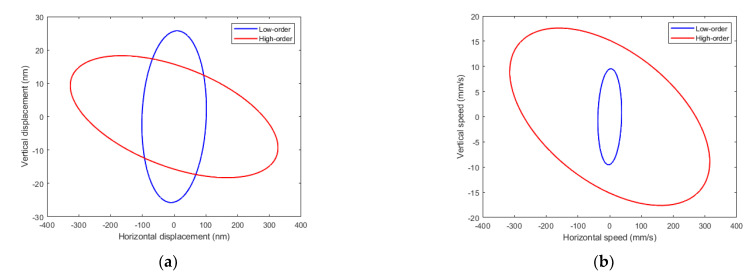
Simulated (**a**) displacement and (**b**) speed of the trajectory of the tip of the legs situated at the left side of the robot, for both the high-order and the low-order combinations.

In order to give an explanation to the effect of mode couple on the speed shown in Figure 6, we might consider that the horizontal displacement at the tip of the legs is proportional to both the amplitude of the vertical displacement at the supporting platform and the length of the legs [23]. According to Figure 2, a higher vertical displacement was expected for the low-order combination. Besides, the length of the legs is longer for the low-order combination than for the high-order combination: 1.2 versus 0.5 mm. Therefore, both factors would lead to a greater displacement of the tip of the legs with the low-order combination. However, another parameter should be taken into account to explain the higher speed, and this is the frequency of actuation. In the case of an ideal elliptical trajectory with harmonic oscillations, the speed of the leg would be proportional to both the amplitude of displacement and the frequency. The frequency of the high-order modes was more than twice the low-order combination, and this had a major contribution to the better performance measured. In order to clarify the effect of all these variables, the finite element model mentioned before in Section 2.1 was used. Actuation for TW generation used the mid-frequency between modes and 90° phase shift. The amplitude of the voltage applied was 10 V. Figure 7 shows the trajectory obtained for the tip of the legs situated at the left side of the robot, for both the low- and the high-order combinations. Figure 7a corresponds to the displacement of the tip and Figure 7b to the speed along the trajectory of the tip. A higher vertical displacement can be observed for the low-order combination, as predicted by means of the 1D model presented before. Regarding the speed, a clear higher value was obtained for the horizontal speed of the high-order combination, responsible for the locomotion of the robot, in qualitative agreement with the experimental results of Figure 6. Similar speed results were obtained for the legs situated at the right side of the robot.

**Figure 8:**
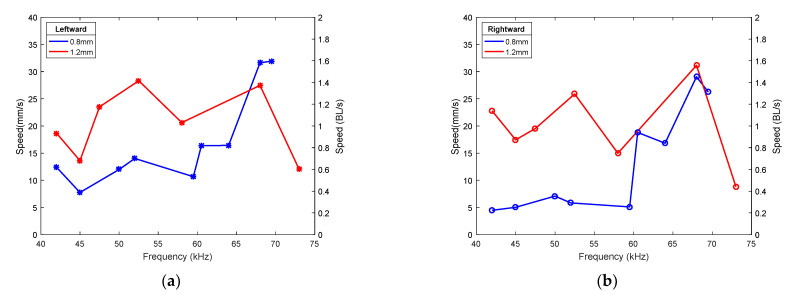
Speed of the TW robot with the low-order combination with 0.8 and 1.2 mm long legs for (**a**) leftward movement, and (**b**) rightward movement.

An additional contribution of this work was to assume the legs as active elements, instead of passive. The idea was to couple the TW on the supporting platform and the first flexural vibration of the legs, with the purpose of affecting the overall performance of the robot. For this study, a range of frequencies between the frequencies of vibration of the two mixed modes was evaluated. For each frequency, the phase shift between the signals applied to the patches was tuned in order to search for the best possible speed in both directions. The possibility of generating the TW by adjusting the values of the frequency of actuation and the phase shift was already mentioned in the design section. A positive effect of the plate–leg coupling could translate into a significant increase in the speed of motion. Figure 8 shows the results for the low-order combination, corresponding to 1.2 mm long legs. Both directions were considered and the voltage applied was 40 Vpp. For comparison purposes, the measurements for a different length of the legs (0.8 mm) were also included. For such a length, the resonance between the plate and the leg was avoided as the first vibration of the leg was estimated at 90.6 kHz. According to Figure 8, a higher speed of motion could be achieved in the low-frequency region of the covered range for the 1.2 mm long legs, but such an increase was not considered enough to ensure a performance improvement related to the coupling of the TW and the vibration of the legs. A similar conclusion was obtained for the high-order combination.

### 4.2. SW-Based Locomotion Results

**Figure 9:**
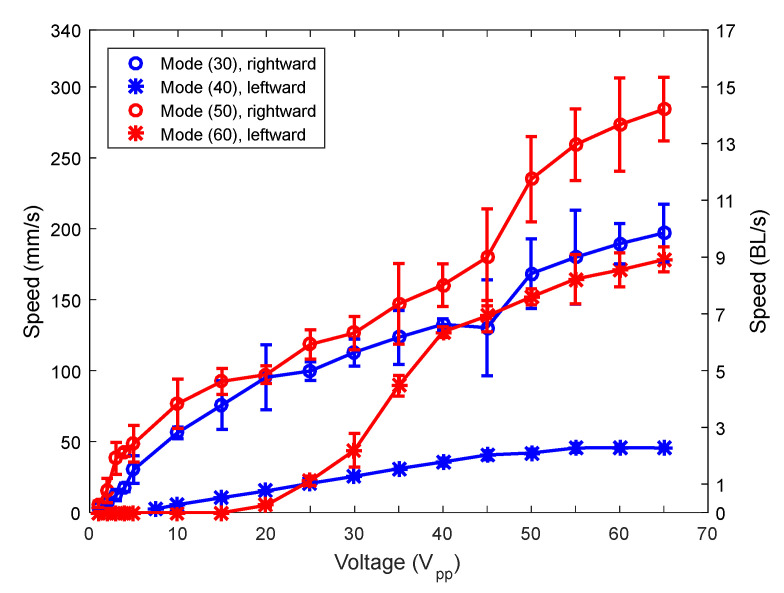
Speed of the robots versus applied voltage for mode (30), mode (40), mode (50) and mode (60).

Next the SW-based locomotion performance was considered. Figure 9 shows the results for the same two samples as in the previous section. Symbols represent the average of five measurements and are joined with lines for guidance purposes. The so-called low-order combination device allowed us to actuate modes (30) and (40); and the high-order combination, modes (50) and (60). The first point to notice is that the speed that can be attained with the SW-based approach is higher than with the TW-based approach. This was expected, as the actuation at the resonance frequency of the vibration mode implies a much higher displacement for the legs than at the mid-frequency between modes that was used for the TW-based approach. Secondly, it is worth pointing out the achievement of speeds as high as 14 BL/s. This value compares with state-of-the-art reports for 1 mg robots with magnetic actuation [18]. In our case, we managed to reach such a value of speed for a mass that was about 250 times higher, with the use of piezoelectric materials that allowed for an all-electrical scheme, and with applied voltages well below 100 V, which might facilitate the implementation of an untethered robot with an integrated driving signal. Video S3, included with the supplementary information, shows the motion in both directions due to the modes (30) and (40). Video S4 is the equivalent for modes (50) and (60).

A final comment should pay attention to the asymmetry that can be observed depending on the direction of movement, especially evident for mode (30). This could be associated with the tight margin for the location of the legs to achieve bidirectional movement for the SW-based approach. If we go back to Figure 3, the positions of the legs were marked with the dashed rectangles, being narrower than 2 mm in some cases. As can be seen, a slight deviation of the leg position from the center of the rectangle might result in a leg at a node or a peak in the modal displacement, locations where the effectiveness of the leg to induce locomotion is null despite the excitation at the resonance. As a result, the implemented position of the legs was critical for the sought bidirectional motion, making it difficult to ensure the same performance in both directions, as in the case of the asymmetries observed in Figure 9. The difficulties related to the manual position of the legs should be overcome by fabrication protocols that allow for monolithic structures comprising both the supporting platform and the legs, such as, for example, 3D printing techniques.

**Figure 10:**
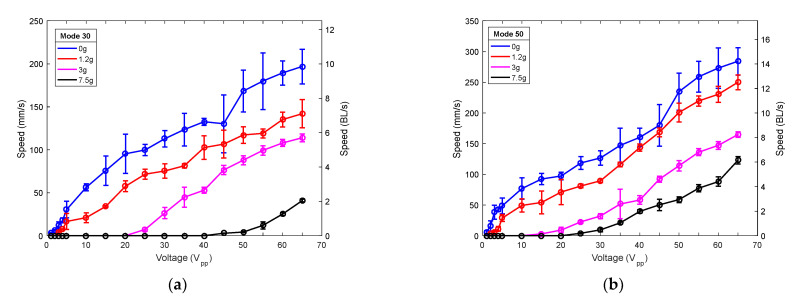
Speed of (**a**) the (30) mode-actuated robot and (**b**) the (50) mode-actuated robot, versus applied voltage for different masses: no load (blue), 1.2 g (red), 3 g (pink), and 7.5 g (black).

The effect of mass loading on the performance of the robots was also investigated. Figure 10 again shows the speed versus applied voltage, but for different loading masses, due to the actuation of the mode (30) and the mode (50). Symbols represent the average of five measurements and are joined with lines for guidance purposes. The robots could carry a mass of 7.5 g, which is 30 times its own weight, at a speed of 40 mm/s for mode (30) and of 122 mm/s for mode (50), both at the maximum applied voltage. These results demonstrated the potentiality of the devices to include electronic circuits on board.

**Figure 11:**
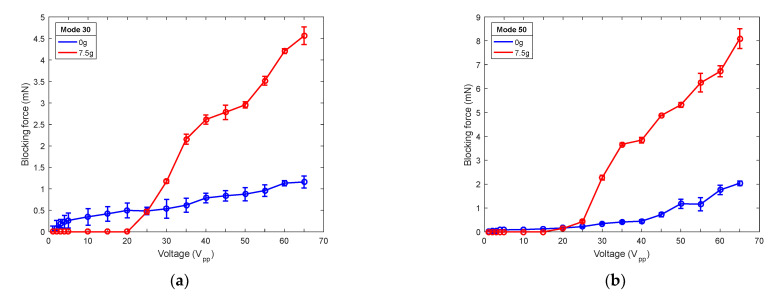
Blocking force of (**a**) the (30) mode-actuated robot and (**b**) the (50) mode-actuated robot, versus applied voltage for different masses: no load mass (blue), 7.5 g (red).

To complete the characterization of the robot, Figure 11 displays the blocking force under different mass loadings. Symbols represent the average of five measurements and are joined with lines for guidance purposes. The force was measured while the robot contacted a force sensor (Honeywell FSG Series) with the actuation voltage applied. As expected, the blocking force increased as the mass loading increased [35].

## 5. Conclusions

This work covers the analysis of the bidirectional motion of miniature mm-sized robots based on a glass platform featuring 3D-printed legs. The attachment of piezoelectric patches allowed for the generation of either SW or TW waves that, combined with the legs, induced the locomotion of the robots. SW-based locomotion resulted in a better performance, as expected, due to the excitation at the resonance frequency of the vibration mode, but the reduced size of the regions where the legs are attached to attain bidirectional motion introduced a practical limitation in the actual implementation. Despite the fabrication difficulties, speed values as high as 14 BL/s could be obtained for a 250 mg robot at an applied voltage of 65 Vpp. Regarding TW-based locomotion, a better performance for the combination of modes of higher order could be observed. Besides, the effect of coupling the first flexural vibration of the legs and the TW of the glass platform was analyzed. The results indicated that such a coupling did not significantly improve the performance of the robots in terms of the speed of locomotion.

## Figures and Tables

**Table 1:** Structural properties of the materials.

	Thickness (mm)	Young Modulus (GPa)	Density (kg/m^3^)
Glass	1	72.9	2653
PZT	0.2	62	6700

**Table 2:** Summary of the parameters to consider for the operation mode of the TW- and the SW-based approaches.

Operation Mode	SW	TW
Patch length	Low-order combination: 8.0 mm High-order combination: 4.9 mm	
Driving frequency	(f1 + f2)/2	Rightward: f1 Leftward: f2
Phase difference	Leftward: 90° Rightward: −90°	Leftward: 0° Rightward: 180°

## Data Availability

Not applicable.

## Supplementary Materials

The following are available online at https://www.mdpi.com/2072-666X/12/2/171/s1, Video S1: Animation of the robot vibration for the TW-based locomotion. Video S2: Animation of the robot vibration for the SW-based locomotion. Video S3: Bidirectional locomotion of a 1.2 mm-legged structure actuated by mode (30), rightward direction, and by mode (40), leftward direction. Video S4: Bidirectional locomotion of a 0.5 mm-legged structure actuated by mode (50), rightward direction, and by mode (60), leftward direction. Some time was required after the rightward movement in order to change the frequency of actuation.
